# Identification of Novel Malaria Transmission-Blocking Vaccine Candidates

**DOI:** 10.3389/fcimb.2021.805482

**Published:** 2021-11-30

**Authors:** Eizo Takashima, Mayumi Tachibana, Masayuki Morita, Hikaru Nagaoka, Bernard N. Kanoi, Takafumi Tsuboi

**Affiliations:** ^1^Division of Malaria Research, Proteo-Science Center, Ehime University, Matsuyama, Japan; ^2^Division of Molecular Parasitology, Proteo-Science Center, Ehime University, Toon, Japan; ^3^Division of Cell-Free Sciences, Proteo-Science Center, Ehime University, Matsuyama, Japan

**Keywords:** immuno-profiling, malaria, *Plasmodium*, reverse vaccinology, transmission-blocking vaccine (TBV), wheat germ cell-free system (WGCFS)

## Abstract

Control measures have significantly reduced malaria morbidity and mortality in the last two decades; however, the downward trends have stalled and have become complicated by the emergence of COVID-19. Significant efforts have been made to develop malaria vaccines, but currently only the RTS,S/AS01 vaccine against *Plasmodium falciparum* has been recommended by the WHO, for widespread use among children in sub-Saharan Africa. The efficacy of RTS,S/AS01 is modest, and therefore the development of more efficacious vaccines is still needed. In addition, the development of transmission-blocking vaccines (TBVs) to reduce the parasite transmission from humans to mosquitoes is required toward the goal of malaria elimination. Few TBVs have reached clinical development, and challenges include low immunogenicity or high reactogenicity in humans. Therefore, novel approaches to accelerate TBV research and development are urgently needed, especially novel TBV candidate discovery. In this mini review we summarize the progress in TBV research and development, novel TBV candidate discovery, and discuss how to accelerate novel TBV candidate discovery.

## Introduction

Malaria continues to be responsible for a substantial global health burden, with 409,000 malarial deaths reported in 2019 (WHO, 2020). From 2000 to 2015, malaria morbidity and mortality were significantly reduced; however, the decreasing trend stalled between 2015 and 2019 and was further complicated by the emergence of COVID-19 (Wang et al., 2020; WHO, 2020). Therefore, the control and eventual eradication of this disease relies on the development of a highly effective malaria vaccine.

Malaria vaccines can be categorized into three groups, each targeting distinct parasite developmental stages: pre-erythrocytic (sporozoite and liver), asexual erythrocytic, and sexual transmission stages. The renewed Malaria Vaccine Technology Roadmap proposes two main objectives by 2030 for the development of new malaria vaccines targeting both *Plasmodium falciparum* and *Plasmodium vivax*: i) vaccines with protective efficacy of at least 75% against clinical malaria, and ii) vaccines that reduce transmission of the parasite (Group, 2013; Moorthy et al., 2013). A leading malaria vaccine RTS,S/AS01 was the first malaria vaccine to enter Phase III clinical trials and shows modest efficacy against clinical falciparum malaria (RTS, 2015) with short durability (White et al., 2015). It is currently being evaluated in a large pilot implementation program in Ghana, Kenya, and Malawi since 2019 (Adepoju, 2019). The vaccine reduced severe malaria by about 30% in the first 2 years of the program (Vogel, 2021). Based on this, the World Health Organization (WHO) is now recommending widespread use of the RTS,S/AS01 malaria vaccine among children in sub-Saharan Africa and in other regions with moderate to high *P. falciparum* malaria transmission (Vogel, 2021).

Since the RTS,S/AS01 vaccine efficacy is modest, the development of more efficacious vaccines is still needed. A number of second-generation malaria vaccines are in clinical trials, such as R21/Matrix-M (Datoo et al., 2021). However, the above mentioned two malaria vaccines are classified as pre-erythrocytic stage vaccines. Therefore, the development of erythrocytic stage vaccines to reduce morbidity and mortality, and transmission-blocking vaccines (TBVs) to reduce parasite transmission from humans to mosquitoes, are required to reach the Roadmap goals.

## Malaria Transmission-Blocking Vaccines (TBVs)

The principle of malaria TBVs is that antibodies against antigen(s) expressed on the sexual stages of the malaria parasite - gametocyte/gamete/zygote/ookinete - reduce the numbers of oocysts in mosquito vectors when fed with gametocytes (Huff et al., 1958; Carter and Chen, 1976; Gwadz, 1976). The advantages of TBVs are summarized as follows (Tsuboi et al., 2003; Miura et al., 2019; Duffy, 2021): i) TBV candidates tend to be less polymorphic than blood- or pre-erythrocytic-stage antigens, presumably due to lower immune pressure driving evolutionary diversity; ii) the absolute number of parasites targeted by TBVs is small, usually <10-100 oocysts per mosquito in nature, and represent a biological bottleneck in the malaria parasite lifecycle; and iii) TBVs might help to prevent the spread of emerging drug-resistant parasites (Dondorp et al., 2009; Balikagala et al., 2021) and future vaccine-escape mutants.

Target antigens include proteins expressed on the surface of gametocytes/gametes/zygotes/ookinetes; such as the characterized proteins P230, P48/45, P28, and P25 (Carter and Kaushal, 1984; Kumar and Carter, 1985; Vermeulen et al., 1985). To initiate vaccine research, the antigens in human malaria parasites were identified in the pre-genomic era; namely, Pfs25 (Kaslow et al., 1988; Kaslow et al., 1994), Pfs28 (Duffy and Kaslow, 1997), Pfs48/45 (Kocken et al., 1993; Outchkourov et al., 2008), and Pfs230 (Williamson et al., 1993; Williamson et al., 1995) from *P. falciparum*; and their orthologs in *P. vivax*, Pvs25 and Pvs28 (Tsuboi et al., 1998; Hisaeda et al., 2000). Soon after whole genome information became accessible, Pvs48/45 (Arevalo-Herrera et al., 2015; Tachibana et al., 2015) and Pvs230 (Tachibana et al., 2012) were also characterized as TBV candidates (**Table 1**).

**Table 1:** Discovery of malaria transmission-blocking vaccine antigens with publication years^a^.

Antigen^b^	Year^c^	Target parasite^d^	Developmental stage^e^	Discovery^f^	Expression system^g^	Reference
**Pre-Genomic Era**						
Pfs25	1988	*Pf*	Zygote/ookinete	Gene	–	(Kaslow et al., 1988)
Pfs25	1994	*Pf*	Zygote/ookinete	TRA	Yeast	(Kaslow et al., 1994)
Pfs28	1997	*Pf*	Zygote/ookinete	Gene/TRA	Yeast	(Duffy and Kaslow, 1997)
Pfs48/45	1993	*Pf*	Gametocyte/gamete	Gene	–	(Kocken et al., 1993)
Pfs48/45	2008	*Pf*	Gametocyte/gamete	TRA	Bacteria	(Outchkourov et al., 2008)
Pfs230	1993	*Pf*	Gametocyte/gamete	Gene	–	(Williamson et al., 1993)
Pfs230	1995	*Pf*	Gametocyte/gamete	TRA	Bacteria	(Williamson et al., 1995)
Pvs25 & Pvs28	1998	*Pv*	Zygote/ookinete	Gene	–	(Tsuboi et al., 1998)
Pvs25 & Pvs28	2000	*Pv*	Zygote/ookinete	TRA	Yeast	(Hisaeda et al., 2000)
**Post-Genomic Era**						
HAP2/GCS1	2008	*Pb*	Gamete	Gene	–	(Hirai et al., 2008; Liu et al., 2008)
HAP2/GCS1	2009	*Pb*	Gamete	TRA	Bacteria	(Blagborough and Sinden, 2009)
HAP2/GCS1	2013	*Pf*	Gamete	TRA	WGCFS	(Miura et al., 2013b)
HAP2/GCS1	2017	*Pb, Pf*	Gamete	TRA	Peptide	(Angrisano et al., 2017)
HAP2/GCS1	2020	*Pv*	Gamete	TRA	Baculovirus	(Qiu et al., 2020)
Pvs230	2012	*Pv*	Gametocyte/gamete	TRA	DNA	(Tachibana et al., 2012)
Pvs48/45, Pvs47	2015	*Pv*	Gametocyte/gamete	TRA	DNA, Bacteria	(Arevalo-Herrera et al., 2015; Tachibana et al., 2015)
Pfs47	2010	*Pf*	Gametocyte/gamete	Gene	–	(van Schaijk et al., 2006)
Pfs47	2018	*Pf*	Gametocyte/gamete	TRA	Bacteria	(Canepa et al., 2018)
AnAPN1	2014	*Pf, Pv*	*Anopheles* midgut	Gene/TRA	*Drosophila* S2	(Armistead et al., 2014)
PbPSOP12	2015	*Pb*	Gamete - ookinete	TRA	BDES	(Sala et al., 2015)
PbPH	2016	*Pb*	Gamete - ookinete	TRA	Bacteria	(Kou et al., 2016)
PbPSOP7, 25 & 26 PbPSOP25	2016 2017	*Pb*	Ookinete	TRA	Bacteria	(Zheng et al., 2016) (Zheng et al., 2017)
Pb51	2017	*Pb*	Gametocyte - ookinete	TRA	Bacteria	(Wang et al., 2017)
Pbg37	2018	*Pb*	Gametocyte - zygote	TRA	Bacteria	(Liu et al., 2018)
PyMiGS	2018 2020	*Py*	Gametocyte/gamete	TRA	WGCFS	(Tachibana et al., 2018a; Tachibana et al., 2018b; Tachibana et al., 2020)
Pb22	2021	*Pb*	Gamete - ookinete	TRA	Bacteria	(Liu et al., 2021)

a Summary of the TBV antigen discovery efforts in which significant TRA has been confirmed.

b Antigen, abbreviated names of TBV antigens.

c Year, year of publication.

d Target parasite, Pf, Plasmodium falciparum; Pv, P. vivax; Pb, P. berghei; Py, P. yoelii.

e Developmental stage, parasite developmental stage(s) in which target the antigen is expressed.

f Discovery, Gene, target gene discovered; TRA, antigens specific antibodies with confirmed transmission reducing/blocking activity identified..

g Expression system, indicates the platform used to express the antigen as either in yeast cells, bacteria, wheat germ cell-free system (WGCFS), Drosophila S2 cells, baculovirus vectored protein expression system or was a synthetic peptide (Peptide). Alternatively, DNA vaccine used as the antigen (DNA). BDES, indicates target antigen was expressed in baculovirus dual expression system.

Researchers have faced a number of difficulties to express TBV antigens with native conformations (Miura et al., 2019), using a variety of protein expression systems (Patel and Tolia, 2021). Antibodies raised against individual antigens needed to be tested in an *ex vivo* efficacy assay; specifically, the standard membrane feeding assay (SMFA) wherein laboratory-reared *Anopheles* mosquitoes are fed on *in vitro* cultured *P. falciparum* gametocytes along with test antisera or purified antibodies, and counts of midgut wall oocysts as a measure of the degree of transmission-blocking activity (Miura et al., 2013a).

## TBV Development Efforts to Date

**Figure 1:**
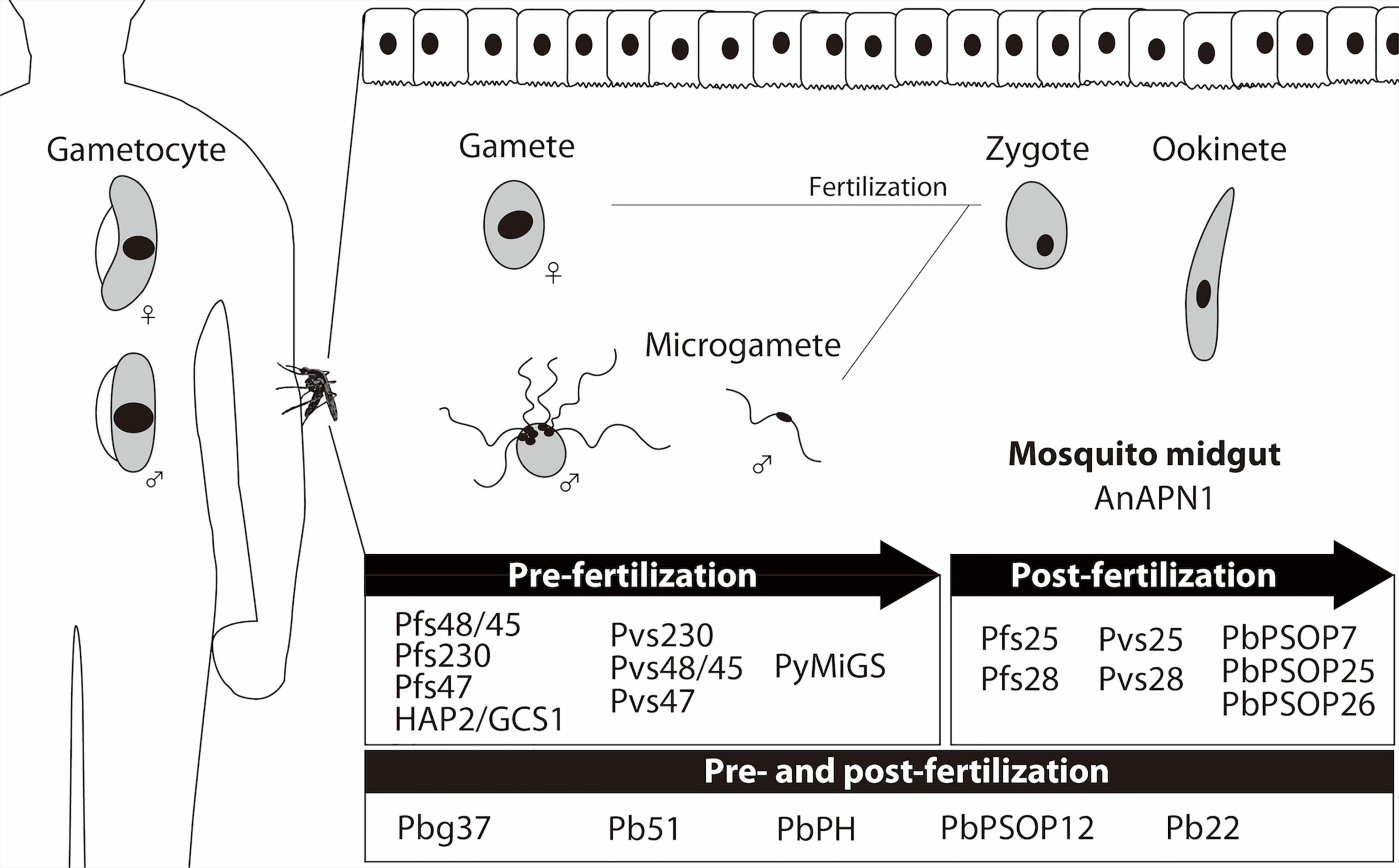
Expression of malaria transmission-blocking vaccine (TBV) target antigens. Sexual developmental stages of malaria parasites in humans (gametocytes) and mosquitoes (gametes, zygotes, and ookinetes) are schematically presented. The TBV candidate antigens (**Table 1**) are categorized as pre-fertilization antigens (mainly expressed in the sexual stages of parasites before fertilization), and post-fertilization antigens (mainly expressed in the sexual stages of parasites after fertilization). Mosquito midgut antigen, AnAPN1, is also presented as a TBV candidate.

After decades of efforts, the most advanced *P. falciparum* TBV antigens in the clinical pipeline remain the first identified antigens: Pfs25 expressed on the surface of zygotes/ookinetes in the mosquito and classified as a post-fertilization antigen, and Pfs48/45 and Pfs230 expressed on the surface of blood-circulating gametocytes and gametes in the mosquito and classified as pre-fertilization antigens. In addition, a mosquito midgut protein, anopheline alanyl aminopeptidase N 1 (AnAPN1) (Armistead et al., 2014), is under development as a TBV candidate in pre-clinical developmental studies (Bender et al., 2021) (**Table 1**, **Figure 1**). As transmission-blocking immunity is mostly antibody-mediated (de Jong et al., 2020), TBV development efforts focus on inducing potent antibodies that are sustained at effective transmission-blocking levels for at least one transmission season. Based on these requirements, extensive efforts towards the clinical development of *P. falciparum* TBVs continue to date. Recently, phase 1 trials of *P. falciparum* TBV based upon Pfs25/Alhydrogel (Alum) have been reported. These studies used Pfs25-EPA: Pfs25 conjugated with a recombinant detoxified ExoProtein A from *Pseudomonas aeruginosa* (EPA), formulated with Alum, and tested in adults in the USA (Talaat et al., 2016) and Mali (Sagara et al., 2018). The vaccine was generally well-tolerated; however, the functional activity of the anti-Pfs25 antibodies induced were modest, and antibody titers decreased rapidly.

To improve functional immunogenicity and durability, the same group performed a phase 1 trial of the pre-fertilization TBV antigen Pfs230 alone or in combination with Pfs25 in USA adults. Pfs25-EPA/Alum and Pfs230D1M [amino acid 542-736 (MacDonald et al., 2016)]-EPA/Alum induced similar serum functional activity in mice, but Pfs230D1M-EPA induced significantly greater activity in rhesus monkeys. In USA adults, two vaccine doses induced functional activity in Pfs230D1M-EPA/Alum volunteers, but no significant activity in Pfs25-EPA vaccine recipients, and combination with Pfs25-EPA did not increase functional activity over Pfs230D1M-EPA alone. The research group concluded that the functional activity of Pfs230D1M-EPA is significantly superior to that of Pfs25-EPA (Healy et al., 2021). For more information about the clinical development of these falciparum TBVs, please refer to two recent review articles (Miura et al., 2019; Duffy, 2021). In addition to the above TBV development efforts, novel TBV candidate discovery is required to accelerate the success in TBV development.

## Post-Genome Novel TBV Candidate Discovery

The goal of identifying new vaccine candidates for both *P. falciparum* and *P. vivax* is aided by whole genome information accessible since 2003 at the malaria genome database (PlasmoDB). The database has been useful to identify vaccine candidates from asexual-blood (Kanoi et al., 2021) and pre-erythrocytic (Bettencourt, 2020) stages. However, the rational selection and prioritization of TBV candidates from the database has yet to be fully explored (Miura et al., 2019). Extensive proteome and transcriptome data from sexual-stage malaria parasites is now available (Lasonder et al., 2016; Meerstein-Kessel et al., 2018) to inform *in silico* TBV candidate discovery. In the following sections we summarize the recent achievements for the discovery of the activities and candidate antigens discovered in the post-genomic era (**Table 1**, **Figure 1**).

## Rodent Malaria Models for Novel TBV Candidate Discovery

Most of the TBV candidates investigated to date have orthologs in rodent malaria parasites, and thus the rodent malaria models are useful for the discovery and characterization of novel TBV candidates. In the last decade several potential TBV candidates have been identified using rodent malaria models. The general strategy of these studies is to select candidate genes from the PlasmoDB according to the following criteria: i) genes must be specifically expressed in sexual-stages; ii) they must share orthologs with human parasites, in particular *P. falciparum* and *P. vivax*; and iii) the presence of a predicted signal peptide with/without transmembrane domain(s) or a GPI-anchor, indicating possible protein export and exposure to inhibitory antibodies. Candidate TBV genes are then expressed in one or more recombinant protein expression systems, followed by immunization of mice. To test efficacy, immunized mice are infected with rodent malaria parasites and then mosquitoes are fed directly on these mice; termed a direct feeding assay. The transmission-blocking activity (TBA) is expressed as a percent reduction of the prevalence of infected mosquitoes; and transmission-reducing activity (TRA) is expressed as a percent reduction of oocyst density.

The majority of such studies were conducted with *P. berghei* rodent parasites because of the ease for genetic manipulation, such as the knockout of candidate genes for functional characterization of novel TBV candidates. Most such activities are listed in **Table 1**, classified in the post-genomic era, and following are descriptions of examples of post-genomic studies.

As the first examples, a group actively working on novel TBV candidate discovery using the *P. berghei* model identified a conserved *P. berghei* protein, PbPH, containing a pleckstrin homology (PH) domain. By indirect immunofluorescence assay (IFA) PbPH localized on the surface of gametes/zygotes/ookinetes. Mice were immunized with recombinant PbPH expressed in *E. coli* and mosquitoes fed on the immunized mice showed a 48% TRA (Kou et al., 2016). Similarly, the same group selected *P. berghei* ookinete-stage proteins, Putative Secreted Ookinete Protein (PbPSOP25), PbPSOP26, and PbPSOP7, for evaluation of their transmission-blocking potentials. Antisera against these bacterially expressed partial recombinant proteins recognized the ookinete surface. Mosquitoes fed on immunized mice showed significant TRAs (60% to 71%) (Zheng et al., 2016). Mice immunized with full-length recombinant PSOP25 expressed in *E. coli* and those receiving passive transfer of an anti-rPSOP25 mAb showed significant TRAs by 66% and 63%, respectively (Zheng et al., 2017).

The conserved *Plasmodium* gene, Pb51, was identified in *P. berghei* through PlasmoDB using gene expression and protein localization criteria. A partial domain of Pb51 was expressed in *E. coli* and mice were immunized. By IFA Pb51 was expressed in schizonts/gametocytes/ookinetes of *P. berghei*. Mice immunized with the recombinant Pb51 showed 55% TRA in direct feeding assays (Wang et al., 2017). Using a similar approach, the same group characterized a protein of 37 kDa preferentially expressed in gametocytes in *P. berghei* (Pbg37). A recombinant Pbg37 (rPbg37) was expressed in bacteria and antibody was generated in mice. IFA showed surface expression of Pbg37 on gametes/zygotes. The rPbg37-immunized mice had a significant TRA (49%) (Liu et al., 2018). Similarly, a gamete/ookinete surface protein of *P. berghei*, Pb22, was identified and recombinant Pb22 was expressed in *E. coli*. The Pb22-immunised mice had a significant TRA (93.5-99.6%) (Liu et al., 2021).

The *P. berghei* ookinete-stage protein, PbPSOP12, was identified based upon annotation as a putative secreted protein and then expressed using the baculovirus dual expression system (BDES). Mouse antibodies against BDES-PbPSOP12 recognized the surface of gametes/ookinetes. Immunization of mice with BDES-PbPSOP12 conferred modest TRA (53%) (Sala et al., 2015).

Our lab has accumulated a number of experiences using the *Plasmodium yoelii* rodent malaria parasite as a suitable model for TBV study; such as the identification of Pfs25 and Pfs28 orthologs in *P. yoelii*, Pys25 and Pys28 (Tsuboi et al., 1997a; Tsuboi et al., 1997b; Tsuboi et al., 1997c). Recently we identified a novel TBV candidate, *P. yoelii* microgamete surface protein (PyMiGS), using a similar approach as mentioned above for the *P. berghei* studies. PyMiGS is a protein expressed in the osmiophilic body of male gametocytes of *P. yoelii* and is translocated to the surface of microgametes. Potent TRA (>99%) was observed in mosquitoes fed on mice passively immunized with antibodies against recombinant full-length PyMiGS expressed using a wheat germ cell-free protein expression system (WGCFS) (Tachibana et al., 2018a). Mice actively immunized with the recombinant full-length PyMiGS conferred >99% TRA using direct mosquito feeding (Tachibana et al., 2018b), and the major epitopes for transmission-blocking antibodies were within the C-terminal region of PyMiGS (Tachibana et al., 2020).

Although the *P. berghei* and *P. yoelii* rodent malaria models are useful to identify novel TBV candidates, results between the models may differ. For example, when we characterized the phenotype of a PyMiGS gene deletion mutant (ΔPyMiGS), the ookinete formation efficiency of ΔPyMiGS was significantly impaired (Tachibana et al., 2018a). Contrary, ookinete formation of the gene deletion mutant of the *P. berghei* ortholog of PyMiGS (PBANKA_1449000) was not impaired (Kehrer et al., 2016). Accordingly, although the usefulness of the rodent models is clear, careful consideration is also required.

Candidates identified in the rodent malaria studies should be evaluated with *P. falciparum* orthologs. For example, a conserved male gamete sterility gene, HAP2/GCS1 (Hapless 2/Generative Cell Specific 1), was initially identified as an essential protein for the fusion of male and female gametes of *P. berghei* (Hirai et al., 2008; Liu et al., 2008). Genetic disruption of the *hap2* locus revealed that parasite fertilization is inhibited, and anti-PbHAP2 sera showed TRA by up to 81% (Blagborough and Sinden, 2009). Mosquitoes fed on mice immunized with PbHAP2 cd loop peptide showed 59% TRA in *P. berghei* and the corresponding TRA in *P. falciparum* was 76% (Angrisano et al., 2017). We also demonstrated strong transmission-blocking activity of mouse antibody against recombinant *P. falciparum* HAP2 protein and concluded the antigen to be a novel TBV candidate (Miura et al., 2013b). Recently, recombinant *P. vivax* HAP2 was expressed in a baculovirus expression system, and rabbit antibody induced significant TRA (40% to 90%) against *P. vivax* field isolates in *Anopheles dirus* (Qiu et al., 2020).

The gametocyte/gamete protein P47 is another example of experimental system-specific differences. When the *p47* gene was disrupted, a strong reduction of female fertility was observed in *P. berghei* (van Dijk et al., 2010), but not in *P. falciparum* (van Schaijk et al., 2006), and anti-Pfs47 mAbs showed no efficacy in *P. falciparum* SMFA (van Schaijk et al., 2006). Similarly, mAbs and polyclonal antibodies against a full-length recombinant Pfs47 protein did not show efficacy in SMFA. However, antibodies against a part of domain 2 in Pfs47 did demonstrate significant TRA (Canepa et al., 2018). Further characterization revealed that when mice were immunized with the full-length protein, almost no antibody was induced against the critical domain 2. Therefore, it is possible that other potential TBV candidates were overlooked in previous studies (Miura et al., 2019); and improvement of antigen design and vaccine formulations with existing TBV candidates, and expansion of the repertoire of novel TBV candidates, are necessary to accelerate TBV development.

## Novel TBV Candidate Discovery Directly Using Human Malaria Parasites

In *P. falciparum* only two studies on genome-wide novel TBV candidate discovery have been reported to date. One is a reverse vaccinology approach by Nikolaeva et al. (Nikolaeva et al., 2020). They identified a panel of potential TBV candidate genes from PlasmoDB by selecting with a sexual-stage specific expression profile. After a logical *in-silico* process to narrow down the candidate list, they expressed 21 recombinant proteins using a human embryonic kidney cell (HEK293) expression system. Twelve proteins were successfully expressed, and mouse antibodies against the recombinant proteins were tested by SMFA. However, none of the novel TBV candidates showed TRA. It is possible that the heterologous human cell expression system resulted in aberrant glycosylation patterns compared with *Plasmodium*, which has a minimal glycosylation machinery, and the resulting antibodies did not recognize native *Plasmodium* protein (Kanoi et al., 2021).

The other is a larger-scale trial of immuno-profiling of naturally occurring antibody-mediated TRA (Stone et al., 2018). Bioinformatically selected 315 proteins were expressed using an *E. coli* cell-free system, and correctly-folded well-characterized recombinant Pfs48/45 and Pfs230 proteins were used as positive controls. They assessed antibody responses in 648 African plasma samples with TRA measured by SMFA, and those with high (≥ 90%, n= 22) or low (< 10%, n=254) TRA were used for the immuno-profiling. Forty-three out of 315 proteins in addition to Pfs230 and Pfs48/45 had significantly higher antibody levels in plasmas with high TRA. After additional consideration on the protein expression levels in gametocytes, and the presence of a signal peptide or a transmembrane domain, 13 out of the 43 proteins were selected as possible TBV candidates. Although the strategy of this work is convincing, to date they have not validated whether any of the 13 novel TBV candidates could induce transmission-blocking antibodies in immunized animals. In addition, since the reacted human antibodies were likely to recognize only linear epitopes of the tested antigens, because the proteins were expressed in *E. coli*, this work may have missed promising candidates which have conformational TRA epitopes/antigens (Miura et al., 2019). Finally, the approach might not identify TBV candidates whose protein expression is solely in the mosquito and not in gametocytes.

Additional gametocyte-specific gene discovery efforts have been published (Ikadai et al., 2013; Chawla et al., 2021; Muthui et al., 2021); although antigen expression, immunization, and TRA assessment of the antibodies are not completed.

## Key Messages to the Novel TBV Candidate Discovery

The clinical development of *P. falciparum* TBV have advanced to Phase 2 clinical trials (Duffy, 2021). However, those efforts have focused only on the leading candidates - Pfs25, Pfs230, and Pfs48/45 - which were identified in the pre-genome era (Miura et al., 2019). To accelerate TBV research and development in the post-genome era, genome-wide discovery of novel TBV candidates by both immuno-profiling and reverse vaccinology approaches are essential. A key message learned from the pioneering post-genome TBV candidate discovery approaches is that it is crucial to select an expression system with the capability of producing large numbers of correctly-folded malaria recombinant proteins, and without artificial glycosylation. We have been using the WGCFS to express a number of high-quality recombinant proteins of both *P. falciparum* and *P. vivax*; and to produce comprehensive genome-wide protein libraries useful for novel malaria vaccine and sero-marker candidate discovery projects (Morita et al., 2017; Kanoi et al., 2018; Longley et al., 2020; Kanoi et al., 2021). Therefore, following genome-wide gametocyte stage protein expression by WGCFS, these proteins can then be used in immuno-profiling approaches using human plasma with known TRA, to identify novel transmission-blocking antigens (Ntege et al., 2017; Miura et al., 2019; Kanoi et al., 2021). To this end it is also essential to obtain well-characterized plasma samples from infected individuals who carry transmission-reducing antibodies.

## Author Contributions

All authors listed have made a substantial, direct, and intellectual contribution to the work and approved it for publication.

## Funding

Some of the work presented here was partially supported by JSPS KAKENHI Grant (JP18H02651, JP20H03481, JP21H02724, JP21K06990, JP21KK0138), Global Health Innovative Technology (GHIT) Fund (grant # G2019-111, G2019-205), and the Takeda Science Foundation. BNK is an EDCTP Fellow under EDCTP2 program supported by the European Union (grant TMA2020CDF-3203-EndPAMAL). The funding sources had no role in study design, collection, analysis, interpretation of data, and publication.

## Conflict of Interest

The authors declare that the research was conducted in the absence of any commercial or financial relationships that could be construed as a potential conflict of interest.

## Publisher’s Note

All claims expressed in this article are solely those of the authors and do not necessarily represent those of their affiliated organizations, or those of the publisher, the editors and the reviewers. Any product that may be evaluated in this article, or claim that may be made by its manufacturer, is not guaranteed or endorsed by the publisher.

## Abbreviations

AnAPN1, anopheline alanyl aminopeptidase N 1; BDES, baculovirus dual expression system; *E*., *Escherichia*; EPA, ExoProtein A from *Pseudomonas aeruginosa*; HAP2/GCS1, Hapless 2/Generative Cell Specific 1; IFA, indirect immunofluorescence assay; MiGS, microgamete surface protein; *P*., *Plasmodium*; PH, pleckstrin homology; PSOP, putative secreted ookinete protein; SMFA, standard membrane feeding assay; TBA, transmission-blocking activity; TBV, transmission-blocking vaccine; TRA, transmission-reducing activity; WGCFS, wheat germ cell-free system; WHO, The World Health Organization.

## References

[B1] Adepoju P. (2019). RTS,S Malaria Vaccine Pilots in Three African Countries. Lancet 393, 1685. doi: 10.1016/S0140-6736(19)30937-7 31034365

[B2] Angrisano F. Sala K. A. Da D. F. Liu Y. Pei J. Grishin N. V.. (2017). Targeting the Conserved Fusion Loop of HAP2 Inhibits the Transmission of Plasmodium Berghei and Falciparum. Cell Rep. 21, 2868–2878. doi: 10.1016/j.celrep.2017.11.024 29212032 PMC5732318

[B3] Arevalo-Herrera M. Vallejo A. F. Rubiano K. Solarte Y. Marin C. Castellanos A.. (2015). Recombinant Pvs48/45 Antigen Expressed in E. Coli Generates Antibodies That Block Malaria Transmission in Anopheles Albimanus Mosquitoes. PloS One 10, e0119335. doi: 10.1371/journal.pone.0119335 25775466 PMC4361554

[B4] Armistead J. S. Morlais I. Mathias D. K. Jardim J. G. Joy J. Fridman A.. (2014). Antibodies to a Single, Conserved Epitope in Anopheles APN1 Inhibit Universal Transmission of Plasmodium Falciparum and Plasmodium Vivax Malaria. Infect. Immun. 82, 818–829. doi: 10.1128/IAI.01222-13 24478095 PMC3911399

[B5] Balikagala B. Fukuda N. Ikeda M. Katuro O. T. Tachibana S. I. Yamauchi M.. (2021). Evidence of Artemisinin-Resistant Malaria in Africa. N. Engl. J. Med. 385, 1163–1171. doi: 10.1056/NEJMoa2101746 34551228

[B6] Bender N. G. Khare P. Martinez J. Tweedell R. E. Nyasembe V. O. Lopez-Gutierrez B.. (2021). Immunofocusing Humoral Immunity Potentiates the Functional Efficacy of the AnAPN1 Malaria Transmission-Blocking Vaccine Antigen. NPJ Vaccines 6, 49. doi: 10.1038/s41541-021-00309-4 33824336 PMC8024329

[B7] Bettencourt P. (2020). Current Challenges in the Identification of Pre-Erythrocytic Malaria Vaccine Candidate Antigens. Front. Immunol. 11:190. doi: 10.3389/fimmu.2020.00190 32153565 PMC7046804

[B8] Blagborough A. M. Sinden R. E. (2009). Plasmodium Berghei HAP2 Induces Strong Malaria Transmission-Blocking Immunity *In Vivo* and *In Vitro*. Vaccine 27, 5187–5194. doi: 10.1016/j.vaccine.2009.06.069 19596419

[B9] Canepa G. E. Molina-Cruz A. Yenkoidiok-Douti L. Calvo E. Williams A. E. Burkhardt M.. (2018). Antibody Targeting of a Specific Region of Pfs47 Blocks Plasmodium Falciparum Malaria Transmission. NPJ Vaccines 3, 26. doi: 10.1038/s41541-018-0065-5 30002917 PMC6039440

[B10] Carter R. Chen D. H. (1976). Malaria Transmission Blocked by Immunisation With Gametes of the Malaria Parasite. Nature 263, 57–60. doi: 10.1038/263057a0 986561

[B11] Carter R. Kaushal D. C. (1984). Characterization of Antigens on Mosquito Midgut Stages of Plasmodium Gallinaceum. III. Changes in Zygote Surface Proteins During Transformation to Mature Ookinete. Mol. Biochem. Parasitol 13, 235–241. doi: 10.1016/0166-6851(84)90116-6 6151115

[B12] Chawla J. Oberstaller J. Adams J. H. (2021). Targeting Gametocytes of the Malaria Parasite Plasmodium Falciparum in a Functional Genomics Era: Next Steps. Pathogens 10:346. doi: 10.3390/pathogens10030346 33809464 PMC7999360

[B13] Datoo M. S. Natama M. H. Some A. Traore O. Rouamba T. Bellamy D.. (2021). Efficacy of a Low-Dose Candidate Malaria Vaccine, R21 in Adjuvant Matrix-M, With Seasonal Administration to Children in Burkina Faso: A Randomised Controlled Trial. Lancet 397, 1809–1818. doi: 10.1016/S0140-6736(21)00943-0 33964223 PMC8121760

[B14] De Jong R. M. Tebeje S. K. Meerstein-Kessel L. Tadesse F. G. Jore M. M. Stone W.. (2020). Immunity Against Sexual Stage Plasmodium Falciparum and Plasmodium Vivax Parasites. Immunol. Rev. 293, 190–215. doi: 10.1111/imr.12828 31840844 PMC6973022

[B15] Dondorp A. M. Nosten F. Yi P. Das D. Phyo A. P. Tarning J.. (2009). Artemisinin Resistance in Plasmodium Falciparum Malaria. N. Engl. J. Med. 361, 455–467. doi: 10.1056/NEJMoa0808859 19641202 PMC3495232

[B16] Duffy P. E. (2021). Transmission-Blocking Vaccines: Harnessing Herd Immunity for Malaria Elimination. Expert Rev. Vaccines 20, 185–198. doi: 10.1080/14760584.2021.1878028 33478283 PMC11127254

[B17] Duffy P. E. Kaslow D. C. (1997). A Novel Malaria Protein, Pfs28, and Pfs25 are Genetically Linked and Synergistic as Falciparum Malaria Transmission-Blocking Vaccines. Infect. Immun. 65, 1109–1113. doi: 10.1128/iai.65.3.1109-1113.1997 9038325 PMC175097

[B18] Group M. V. F. (2013). Malaria Vaccine Technology Roadmap (Geneva, Switzerland: WHO Press).

[B19] Gwadz R. W. (1976). Successful Immunization Against the Sexual Stages of Plasmodium Gallinaceum. Science 193, 1150–1151. doi: 10.1126/science.959832 959832

[B20] Healy S. A. Anderson C. Swihart B. J. Mwakingwe A. Gabriel E. E. Decederfelt H.. (2021). Pfs230 Yields Higher Malaria Transmission-Blocking Vaccine Activity Than Pfs25 in Humans But Not Mice. J. Clin. Invest. 131, e146221. doi: 10.1172/JCI146221 PMC8011888 33561016

[B21] Hirai M. Arai M. Mori T. Miyagishima S. Y. Kawai S. Kita K.. (2008). Male Fertility of Malaria Parasites Is Determined by GCS1, a Plant-Type Reproduction Factor. Curr. Biol. 18, 607–613. doi: 10.1016/j.cub.2008.03.045 18403203

[B22] Hisaeda H. Stowers A. W. Tsuboi T. Collins W. E. Sattabongkot J. S. Suwanabun N.. (2000). Antibodies to Malaria Vaccine Candidates Pvs25 and Pvs28 Completely Block the Ability of Plasmodium Vivax to Infect Mosquitoes. Infect. Immun. 68, 6618–6623. doi: 10.1128/iai.68.12.6618-6623.2000 11083773 PMC97758

[B23] Huff C. G. Marchbank D. F. Shiroishi T. (1958). Changes in Infectiousness of Malarial Gametocytes. II. Analysis of the Possible Causative Factors. Exp. Parasitol 7, 399–417. doi: 10.1016/0014-4894(58)90036-5 13562104

[B24] Ikadai H. Shaw Saliba K. Kanzok S. M. Mclean K. J. Tanaka T. Q. Cao J.. (2013). Transposon Mutagenesis Identifies Genes Essential for Plasmodium Falciparum Gametocytogenesis. Proc. Natl. Acad. Sci. U.S.A. 110, E1676–E1684. doi: 10.1073/pnas.1217712110 23572579 PMC3645567

[B25] Kanoi B. N. Nagaoka H. Morita M. Tsuboi T. Takashima E. (2021). Leveraging the Wheat Germ Cell-Free Protein Synthesis System to Accelerate Malaria Vaccine Development. Parasitol Int. 80:102224. doi: 10.1016/j.parint.2020.102224 33137499

[B26] Kanoi B. N. Nagaoka H. Morita M. White M. T. Palacpac N. M. Q. Ntege E. H.. (2018). Comprehensive Analysis of Antibody Responses to Plasmodium Falciparum Erythrocyte Membrane Protein 1 Domains. Vaccine 36, 6826–6833. doi: 10.1016/j.vaccine.2018.08.058 30262245

[B27] Kaslow D. C. Bathurst I. C. Lensen T. Ponnudurai T. Barr P. J. Keister D. B. (1994). Saccharomyces Cerevisiae Recombinant Pfs25 Adsorbed to Alum Elicits Antibodies That Block Transmission of Plasmodium Falciparum. Infect. Immun. 62, 5576–5580. doi: 10.1128/iai.62.12.5576-5580.1994 7960139 PMC303304

[B28] Kaslow D. C. Quakyi I. A. Syin C. Raum M. G. Keister D. B. Coligan J. E.. (1988). A Vaccine Candidate From the Sexual Stage of Human Malaria That Contains EGF-Like Domains. Nature 333, 74–76. doi: 10.1038/333074a0 3283563

[B29] Kehrer J. Frischknecht F. Mair G. R. (2016). Proteomic Analysis of the Plasmodium Berghei Gametocyte Egressome and Vesicular bioID of Osmiophilic Body Proteins Identifies Merozoite TRAP-Like Protein (MTRAP) as an Essential Factor for Parasite Transmission. Mol. Cell Proteomics 15, 2852–2862. doi: 10.1074/mcp.M116.058263 27371728 PMC5013303

[B30] Kocken C. H. Jansen J. Kaan A. M. Beckers P. J. Ponnudurai T. Kaslow D. C.. (1993). Cloning and Expression of the Gene Coding for the Transmission Blocking Target Antigen Pfs48/45 of Plasmodium Falciparum. Mol. Biochem. Parasitol 61, 59–68. doi: 10.1016/0166-6851(93)90158-T 8259133

[B31] Kou X. Zheng W. Du F. Liu F. Wang M. Fan Q.. (2016). Characterization of a Plasmodium Berghei Sexual Stage Antigen PbPH as a New Candidate for Malaria Transmission-Blocking Vaccine. Parasit Vectors 9, 190. doi: 10.1186/s13071-016-1459-8 27038925 PMC4818878

[B32] Kumar N. Carter R. (1985). Biosynthesis of Two Stage-Specific Membrane Proteins During Transformation of Plasmodium Gallinaceum Zygotes Into Ookinetes. Mol. Biochem. Parasitol 14, 127–139. doi: 10.1016/0166-6851(85)90032-5 4039406

[B33] Lasonder E. Rijpma S. R. Van Schaijk B. C. Hoeijmakers W. A. Kensche P. R. Gresnigt M. S.. (2016). Integrated Transcriptomic and Proteomic Analyses of P. Falciparum Gametocytes: Molecular Insight Into Sex-Specific Processes and Translational Repression. Nucleic Acids Res. 44, 6087–6101. doi: 10.1093/nar/gkw536 27298255 PMC5291273

[B34] Liu F. Li L. Zheng W. He Y. Wang Y. Zhu X.. (2018). Characterization of Plasmodium Berghei Pbg37 as Both a Pre- and Postfertilization Antigen With Transmission-Blocking Potential. Infect. Immun. 86, e00785–17. doi: 10.1128/IAI.00785-17 PMC6056874 29866905

[B35] Liu Y. Tewari R. Ning J. Blagborough A. M. Garbom S. Pei J.. (2008). The Conserved Plant Sterility Gene HAP2 Functions After Attachment of Fusogenic Membranes in Chlamydomonas and Plasmodium Gametes. Genes Dev. 22, 1051–1068. doi: 10.1101/gad.1656508 18367645 PMC2335326

[B36] Liu F. Yang F. Wang Y. Hong M. Zheng W. Min H.. (2021). A Conserved Malaria Parasite Antigen Pb22 Plays a Critical Role in Male Gametogenesis in Plasmodium Berghei. Cell Microbiol. 23, e13294. doi: 10.1111/cmi.13294 33222390 PMC8194029

[B37] Longley R. J. White M. T. Takashima E. Brewster J. Morita M. Harbers M.. (2020). Development and Validation of Serological Markers for Detecting Recent Plasmodium Vivax Infection. Nat. Med. 26, 741–749. doi: 10.1038/s41591-020-0841-4 32405064

[B38] Macdonald N. J. Nguyen V. Shimp R. Reiter K. Herrera R. Burkhardt M.. (2016). Structural and Immunological Characterization of Recombinant 6-Cysteine Domains of the Plasmodium Falciparum Sexual Stage Protein Pfs230. J. Biol. Chem. 291, 19913–19922. doi: 10.1074/jbc.M116.732305 27432885 PMC5025679

[B39] Meerstein-Kessel L. van der Lee R. Stone W. Lanke K. Baker D. A. Alano P.. (2018). Probabilistic Data Integration Identifies Reliable Gametocyte-Specific Proteins and Transcripts in Malaria Parasites. Sci. Rep. 8, 410. doi: 10.1038/s41598-017-18840-7 29323249 PMC5765010

[B40] Miura K. Deng B. Tullo G. Diouf A. Moretz S. E. Locke E.. (2013a). Qualification of Standard Membrane-Feeding Assay With Plasmodium Falciparum Malaria and Potential Improvements for Future Assays. PloS One 8, e57909. doi: 10.1371/journal.pone.0057909 23483940 PMC3590281

[B41] Miura K. Tachibana M. Takashima E. Morita M. Kanoi B. N. Nagaoka H.. (2019). Malaria Transmission-Blocking Vaccines: Wheat Germ Cell-Free Technology can Accelerate Vaccine Development. Expert Rev. Vaccines 18, 1017–1027. doi: 10.1080/14760584.2019.1674145 31566026 PMC11000147

[B42] Miura K. Takashima E. Deng B. Tullo G. Diouf A. Moretz S. E.. (2013b). Functional Comparison of Plasmodium Falciparum Transmission-Blocking Vaccine Candidates by the Standard Membrane-Feeding Assay. Infect. Immun. 81, 4377–4382. doi: 10.1128/IAI.01056-13 24042109 PMC3838000

[B43] Moorthy V. S. Newman R. D. Okwo-Bele J. M. (2013). Malaria Vaccine Technology Roadmap. Lancet 382, 1700–1701. doi: 10.1016/S0140-6736(13)62238-2 24239252

[B44] Morita M. Takashima E. Ito D. Miura K. Thongkukiatkul A. Diouf A.. (2017). Immunoscreening of Plasmodium Falciparum Proteins Expressed in a Wheat Germ Cell-Free System Reveals a Novel Malaria Vaccine Candidate. Sci. Rep. 7:46086. doi: 10.1038/srep46086 28378857 PMC5380959

[B45] Muthui M. K. Takashima E. Omondi B. R. Kinya C. Muasya W. I.. (2021). Characterization of Naturally Acquired Immunity to a Panel of Antigens Expressed in Mature P. Falciparum Gametocytes. Front. Cell Infect. Microbiol. 11, 774537. doi: 10.3389/fcimb.2021.774537 34869075 PMC8633105

[B46] Nikolaeva D. Illingworth J. J. Miura K. Alanine D. G. W. Brian I. J. Li Y.. (2020). Functional Characterization and Comparison of Plasmodium Falciparum Proteins as Targets of Transmission-Blocking Antibodies. Mol. Cell Proteomics 19, 155–166. doi: 10.1074/mcp.RA117.000036 29089373 PMC6944241

[B47] Ntege E. H. Takashima E. Morita M. Nagaoka H. Ishino T. Tsuboi T. (2017). Blood-Stage Malaria Vaccines: Post-Genome Strategies for the Identification of Novel Vaccine Candidates. Expert Rev. Vaccines 16, 769–779. doi: 10.1080/14760584.2017.1341317 28604122

[B48] Outchkourov N. S. Roeffen W. Kaan A. Jansen J. Luty A. Schuiffel D.. (2008). Correctly Folded Pfs48/45 Protein of Plasmodium Falciparum Elicits Malaria Transmission-Blocking Immunity in Mice. Proc. Natl. Acad. Sci. U.S.A. 105, 4301–4305. doi: 10.1073/pnas.0800459105 18332422 PMC2393789

[B49] Patel P. N. Tolia N. (2021). Structural Vaccinology of Malaria Transmission-Blocking Vaccines. Expert Rev. Vaccines 20, 199–214. doi: 10.1080/14760584.2021.1873135 33430656 PMC11077433

[B50] Qiu Y. Zhao Y. Liu F. Ye B. Zhao Z. Thongpoon S.. (2020). Evaluation of Plasmodium Vivax HAP2 as a Transmission-Blocking Vaccine Candidate. Vaccine 38, 2841–2848. doi: 10.1016/j.vaccine.2020.02.011 32093983 PMC7217802

[B51] Rts, S.C.T.P. (2015). Efficacy and Safety of RTS,S/AS01 Malaria Vaccine With or Without a Booster Dose in Infants and Children in Africa: Final Results of a Phase 3, Individually Randomised, Controlled Trial. Lancet 386, 31–45. doi: 10.1016/S0140-6736(15)60721-8 25913272 PMC5626001

[B52] Sagara I. Healy S. A. Assadou M. H. Gabriel E. E. Kone M. Sissoko K.. (2018). Safety and Immunogenicity of Pfs25H-EPA/Alhydrogel, a Transmission-Blocking Vaccine Against Plasmodium Falciparum: A Randomised, Double-Blind, Comparator-Controlled, Dose-Escalation Study in Healthy Malian Adults. Lancet Infect. Dis. 18, 969–982. doi: 10.1016/S1473-3099(18)30344-X 30061051 PMC6287938

[B53] Sala K. A. Nishiura H. Upton L. M. Zakutansky S. E. Delves M. J. Iyori M.. (2015). The Plasmodium Berghei Sexual Stage Antigen PSOP12 Induces Anti-Malarial Transmission Blocking Immunity Both *In Vivo* and *In Vitro*. Vaccine 33, 437–445. doi: 10.1016/j.vaccine.2014.11.038 25454088

[B54] Stone W. J. R. Campo J. J. Ouedraogo A. L. Meerstein-Kessel L. Morlais I. Da D.. (2018). Unravelling the Immune Signature of Plasmodium Falciparum Transmission-Reducing Immunity. Nat. Commun. 9, 558. doi: 10.1038/s41467-017-02646-2 29422648 PMC5805765

[B55] Tachibana M. Baba M. Takashima E. Tsuboi T. Torii M. Ishino T. (2020). The C-Terminal Region of the Plasmodium Yoelii Microgamete Surface Antigen PyMiGS Induces Potent Anti-Malarial Transmission-Blocking Immunity in Mice. Vaccine 38, 3129–3136. doi: 10.1016/j.vaccine.2020.02.058 32147299

[B56] Tachibana M. Ishino T. Takashima E. Tsuboi T. Torii M. (2018a). A Male Gametocyte Osmiophilic Body and Microgamete Surface Protein of the Rodent Malaria Parasite Plasmodium Yoelii (PyMiGS) Plays a Critical Role in Male Osmiophilic Body Formation and Exflagellation. Cell Microbiol. 20, e12821. doi: 10.1111/cmi.12821 29316140 PMC5901010

[B57] Tachibana M. Ishino T. Tsuboi T. Torii M. (2018b). The Plasmodium Yoelii Microgamete Surface Antigen (PyMiGS) Induces Anti-Malarial Transmission Blocking Immunity That Reduces Microgamete Motility/Release From Activated Male Gametocytes. Vaccine 36, 7463–7471. doi: 10.1016/j.vaccine.2018.10.067 30420038

[B58] Tachibana M. Sato C. Otsuki H. Sattabongkot J. Kaneko O. Torii M.. (2012). Plasmodium Vivax Gametocyte Protein Pvs230 Is a Transmission-Blocking Vaccine Candidate. Vaccine 30, 1807–1812. doi: 10.1016/j.vaccine.2012.01.003 22245309

[B59] Tachibana M. Suwanabun N. Kaneko O. Iriko H. Otsuki H. Sattabongkot J.. (2015). Plasmodium Vivax Gametocyte Proteins, Pvs48/45 and Pvs47, Induce Transmission-Reducing Antibodies by DNA Immunization. Vaccine 33, 1901–1908. doi: 10.1016/j.vaccine.2015.03.008 25765968

[B60] Talaat K. R. Ellis R. D. Hurd J. Hentrich A. Gabriel E. Hynes N. A.. (2016). Safety and Immunogenicity of Pfs25-EPA/Alhydrogel(R), a Transmission Blocking Vaccine Against Plasmodium Falciparum: An Open Label Study in Malaria Naive Adults. PloS One 11, e0163144. doi: 10.1371/journal.pone.0163144 27749907 PMC5066979

[B61] Tsuboi T. Cao Y. M. Hitsumoto Y. Yanagi T. Kanbara H. Torii M. (1997a). Two Antigens on Zygotes and Ookinetes of Plasmodium Yoelii and Plasmodium Berghei That are Distinct Targets of Transmission-Blocking Immunity. Infect. Immun. 65, 2260–2264. doi: 10.1128/iai.65.6.2260-2264.1997 9169761 PMC175313

[B62] Tsuboi T. Cao Y. M. Kaslow D. C. Shiwaku K. Torii M. (1997b). Primary Structure of a Novel Ookinete Surface Protein From Plasmodium Berghei. Mol. Biochem. Parasitol 85, 131–134. doi: 10.1016/S0166-6851(96)02821-6 9108555

[B63] Tsuboi T. Kaslow D. C. Cao Y. M. Shiwaku K. Torii M. (1997c). Comparison of Plasmodium Yoelii Ookinete Surface Antigens With Human and Avian Malaria Parasite Homologues Reveals Two Highly Conserved Regions. Mol. Biochem. Parasitol 87, 107–111. doi: 10.1016/S0166-6851(97)00049-2 9233679

[B64] Tsuboi T. Kaslow D. C. Gozar M. M. Tachibana M. Cao Y. M. Torii M. (1998). Sequence Polymorphism in Two Novel Plasmodium Vivax Ookinete Surface Proteins, Pvs25 and Pvs28, That Are Malaria Transmission-Blocking Vaccine Candidates. Mol. Med. 4, 772–782. doi: 10.1007/BF03401770 9990863 PMC2230397

[B65] Tsuboi T. Tachibana M. Kaneko O. Torii M. (2003). Transmission-Blocking Vaccine of Vivax Malaria. Parasitol Int. 52, 1–11. doi: 10.1016/s1383-5769(02)00037-5 12543142

[B66] Van Dijk M. R. Van Schaijk B. C. Khan S. M. Van Dooren M. W. Ramesar J. Kaczanowski S.. (2010). Three Members of the 6-Cys Protein Family of Plasmodium Play a Role in Gamete Fertility. PloS Pathog. 6, e1000853. doi: 10.1371/journal.ppat.1000853 20386715 PMC2851734

[B67] Van Schaijk B. C. Van Dijk M. R. Van De Vegte-Bolmer M. Van Gemert G. J. Van Dooren M. W. Eksi S.. (2006). Pfs47, Paralog of the Male Fertility Factor Pfs48/45, Is a Female Specific Surface Protein in Plasmodium Falciparum. Mol. Biochem. Parasitol 149, 216–222. doi: 10.1016/j.molbiopara.2006.05.015 16824624

[B68] Vermeulen A. N. Ponnudurai T. Beckers P. J. Verhave J. P. Smits M. A. Meuwissen J. H. (1985). Sequential Expression of Antigens on Sexual Stages of Plasmodium Falciparum Accessible to Transmission-Blocking Antibodies in the Mosquito. J. Exp. Med. 162, 1460–1476. doi: 10.1084/jem.162.5.1460 2865324 PMC2187939

[B69] Vogel G. (2021). WHO Gives First Malaria Vaccine the Green Light. Science 374, 245–246. doi: 10.1126/science.acx9344 34648307

[B70] Wang J. Xu C. Wong Y. K. He Y. Adegnika A. A. Kremsner P. G.. (2020). Preparedness Is Essential for Malaria-Endemic Regions During the COVID-19 Pandemic. Lancet 395, 1094–1096. doi: 10.1016/S0140-6736(20)30561-4 32192582 PMC7158917

[B71] Wang J. Zheng W. Liu F. Wang Y. He Y. Zheng L.. (2017). Characterization of Pb51 in Plasmodium Berghei as a Malaria Vaccine Candidate Targeting Both Asexual Erythrocytic Proliferation and Transmission. Malar J. 16, 458. doi: 10.1186/s12936-017-2107-2 29132428 PMC5683326

[B72] White M. T. Verity R. Griffin J. T. Asante K. P. Owusu-Agyei S. Greenwood B.. (2015). Immunogenicity of the RTS,S/AS01 Malaria Vaccine and Implications for Duration of Vaccine Efficacy: Secondary Analysis of Data From a Phase 3 Randomised Controlled Trial. Lancet Infect. Dis. 15, 1450–1458. doi: 10.1016/S1473-3099(15)00239-X 26342424 PMC4655306

[B73] Who (2020). World Malaria Report 2020 (Geneva, Switzerland: WHO Press).

[B74] Williamson K. C. Criscio M. D. Kaslow D. C. (1993). Cloning and Expression of the Gene for Plasmodium Falciparum Transmission-Blocking Target Antigen, Pfs230. Mol. Biochem. Parasitol 58, 355–358. doi: 10.1016/0166-6851(93)90058-6 8479460

[B75] Williamson K. C. Keister D. B. Muratova O. Kaslow D. C. (1995). Recombinant Pfs230, a Plasmodium Falciparum Gametocyte Protein, Induces Antisera That Reduce the Infectivity of Plasmodium Falciparum to Mosquitoes. Mol. Biochem. Parasitol 75, 33–42. doi: 10.1016/0166-6851(95)02507-3 8720173

[B76] Zheng W. Kou X. Du Y. Liu F. Yu C. Tsuboi T.. (2016). Identification of Three Ookinete-Specific Genes and Evaluation of Their Transmission-Blocking Potentials in Plasmodium Berghei. Vaccine 34, 2570–2578. doi: 10.1016/j.vaccine.2016.04.011 27083421 PMC4864593

[B77] Zheng W. Liu F. He Y. Liu Q. Humphreys G. B. Tsuboi T.. (2017). Functional Characterization of Plasmodium Berghei PSOP25 During Ookinete Development and as a Malaria Transmission-Blocking Vaccine Candidate. Parasit Vectors 10:8. doi: 10.1186/s13071-016-1932-4 28057055 PMC5217559

